# An atlas of endogenous DNA double-strand breaks arising during human neural cell fate determination

**DOI:** 10.1038/s41597-022-01508-x

**Published:** 2022-07-12

**Authors:** Roberto Ballarino, Britta A. M. Bouwman, Federico Agostini, Luuk Harbers, Constantin Diekmann, Erik Wernersson, Magda Bienko, Nicola Crosetto

**Affiliations:** 1 Department of Microbiology, Tumor and Cell Biology, Karolinska Institutet, Stockholm, SE-17165 Sweden; 2 Science for Life Laboratory, Tomtebodavägen 23 A, Solna, SE-17165 Sweden; 3 Department of Medical Biochemistry and Biophysics, Karolinska Institutet, Stockholm, SE-17165 Sweden; 4 Human Technopole, Viale Rita Levi-Montalcini 1, 20157 Milan, Italy

**Keywords:** Autism spectrum disorders, Developmental disorders, Double-strand DNA breaks, DNA sequencing

## Abstract

Endogenous DNA double-strand breaks (DSBs) occurring in neural cells have been implicated in the pathogenesis of neurodevelopmental disorders (NDDs). Currently, a genomic map of endogenous DSBs arising during human neurogenesis is missing. Here, we applied in-suspension Breaks Labeling *In Situ* and Sequencing (sBLISS), RNA-Seq, and Hi-C to chart the genomic landscape of DSBs and relate it to gene expression and genome architecture in 2D cultures of human neuroepithelial stem cells (NES), neural progenitor cells (NPC), and post-mitotic neural cells (NEU). Endogenous DSBs were enriched at the promoter and along the gene body of transcriptionally active genes, at the borders of topologically associating domains (TADs), and around chromatin loop anchors. NDD risk genes harbored significantly more DSBs in comparison to other protein-coding genes, especially in NEU cells. We provide sBLISS, RNA-Seq, and Hi-C datasets for each differentiation stage, and all the scripts needed to reproduce our analyses. Our datasets and tools represent a unique resource that can be harnessed to investigate the role of genome fragility in the pathogenesis of NDDs.

## Abstract (summary)

**Table:** 

Measurement(s)	DNA Double Strand Break • Transcriptome • Whole Genome Sequencing • Chromosome conformation capture assay • Tumor Suppressor p53-Binding Protein 1
Technology Type(s)	sBLISS - Genome-wide detection of DNA double-strand breaks by in-suspension breaks labeling *in situ* and sequencing • RNA-seq of total RNA • copy number variation profiling assay • Hi-C • Immunocytochemistry
Factor Type(s)	Human Neural Cell Fate Specification
Sample Characteristic - Organism	Homo sapiens
Sample Characteristic - Environment	cell culture

## Background & Summary

Incorrectly repaired DNA double-strand breaks (DSBs) pose a major threat to genome stability, as they can potentially lead to the formation of mutations and genomic rearrangements that can ultimately cause cancer and other disorders driven by genome instability^1^. Emerging evidence suggests that both transcription and the three-dimensional (3D) genome structure are associated with the formation of endogenous DSBs^2,3^. Transcription-associated DSBs seem to preferentially form around the transcription start site (TSS) of transcriptionally active genes as well as at chromatin loop anchors in proximity of highly transcribed genes, presumably because of the accumulation of DNA torsional stress in these regions, which requires resolution by transient DNA breaks formed by topoisomerases^4,5^.

In mouse neural stem cells, hotspots of endogenous DSBs (so-called recurrent DSB clusters) were previously detected around the TSS of highly transcribed genes involved in general cellular processes and along the gene body of long, neural-specific genes whose human orthologues had been previously implicated in neurodevelopmental disorders (NDDs) such as schizophrenia (SCZ) and autism spectrum disorder (ASD)^6–8^. Interestingly, many of these RDCs were also found in neurotypical human neural stem cells^9^. Moreover, neural stem cells derived from patients with a form of ASD marked by increased susceptibility to replication stress were shown to contain recurrent DSB clusters localized specifically within transcribed genes associated with ASD risk^10^.

**Fig. 1:**
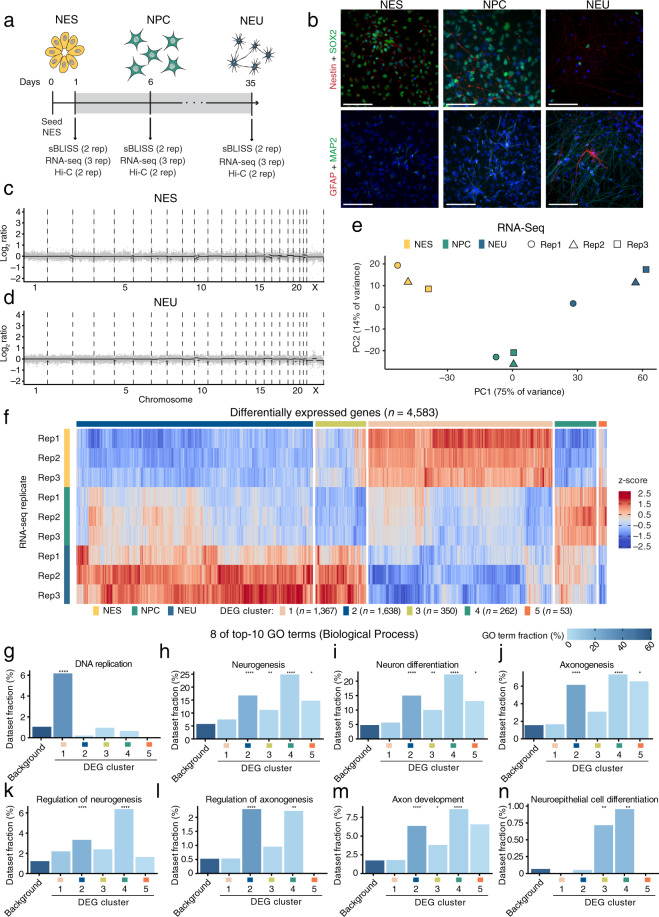
Validation of the model system of human neurogenesis used in this study. (**a**) Timeline of 2D culture of neuroepithelial stem (NES) cell differentiation to neural progenitor cells (NPC) and neuronal (NEU) cells. The rose rectangle indicates the period during which the cells were kept in differentiation conditioning medium (see Methods). Cells were harvested at three timepoints and processed for sBLISS, RNA-Seq, and Hi-C (see Supplementary Table 1 for the list of datasets). Note that different batches of NES cells from different passages (max 5 passages apart) were used for performing multiple replicate (rep) experiments with each technique. (**b**) Maximum z-projections of wide-field epifluorescence microscopy z-stacks showing the expression of different markers of neuronal lineage at the same days (D) of differentiation shown in (**a**). Scale bars, 100 μm. (**c,d**) Genome-wide DNA copy number profiles (100 kb resolution) of NES and NEU cells. Each grey dot represents one 100 kb genomic bin. The black lines indicate the median Log2 ratio between the observed and expected read counts per bin along each chromosome. (**e**) Principal component analysis of the RNA-Seq datasets (Datasets 7–15, see Supplementary Table 1). PC, principal component. Rep, replicate. (**f**) Hierarchical clustering of differentially expressed genes (DEG) between NES, NPC, and NEU cells. Rep, replicate. (**g**–**n**) Enrichment of 8 of the top-10 gene ontology (GO) terms associated with the differentially expressed genes shown in (f), in each of the five clusters shown in (**f**) or in the remaining protein-coding genes (Background).

Despite the emerging evidence linking endogenous DSBs to NDDs, genome-wide maps of DSBs spontaneously arising at different stages of human neurogenesis are currently missing. Such maps would allow correlating the genomic DSB landscape of cells at various stages of neural differentiation with other genomic and epigenomic features, providing clues on how DSBs might form in these cells and how their incorrect repair might contribute to the pathogenesis of NDDs. Towards this goal, we leverage our in-suspension Breaks Labeling *In Situ* and Sequencing (sBLISS) method^11^ together with RNA-Seq and Hi-C^12^ to chart an atlas of endogenous DSBs in relationship with gene expression and 3D genome organization, in 2D cultures of human neuroepithelial stem (NES) cells, neural progenitor cells (NPC) after 6 days of differentiation *in vitro*, and neuronal cells (NEU) after 35 days of differentiation (Fig. 1a). We provide sBLISS^13,14^, RNA-Seq^13^, and Hi-C^13^ datasets from multiple experimental replicates for each differentiation stage (see Supplementary Table 1 for a summary of all the datasets) and all the scripts used to analyze the datasets provided. We show that genome-wide maps of endogenous DSBs are highly correlated between experimental replicates up to 10 kilobases (kb) resolution, and that DSBs are non-uniformly distributed along the genome, in all three cell differentiation stages analyzed. To validate our results, we show that endogenous DSBs are enriched around the promoter of actively transcribed genes as well as around chromatin loop anchors, in agreement with previous observations in non-neural cells and using different DSB detection methods. Lastly, we assess the prevalence of endogenous DSBs in genes previously linked to SCZ and ASD, demonstrating that these genes are significantly more fragile compared to all other protein-coding genes, especially in NEU cells. Our datasets and analytical tools represent a valuable resource for exploring genome fragility during human neurogenesis and investigating how this might contribute to the pathogenesis of NDDs.

## Methods

### Culturing and differentiation of NES cells

We obtained a human, induced pluripotent stem cell (iPSC)-derived, long-term self-renewing NES cell line (AF22) from the human iPS core facility at Karolinska Institutet, where it was previously derived under ethical permit #2012/208-31/3 issued by the local Ethics Review Committee. We cultured these cells following a protocol previously established at the same facility^15^. Briefly, we expanded the cells for up to 12 passages in flasks coated with 0.01% Poly-L-Ornithine (Sigma-Aldrich) and 2 μg/mL L2020 laminin (Sigma-Aldrich). We grew the cells in DMEM-F/12 medium (Thermo Fisher Scientific) supplemented with 1% N2 supplement (Thermo Fisher Scientific) and 0.1% B27 supplement minus Vitamin A (Thermo Fisher Scientific), 10 ng/mL Epidermal Growth Factor (EGF) (Thermo Fisher Scientific), 10 ng/mL basic Fibroblast Growth Factor (bFGF2) (Thermo Fisher Scientific), and 1% Penicillin/Streptomycin (Thermo Fisher Scientific). We hereafter refer to this medium as NES medium. For passaging, we gently detached the cells from the flasks using TrypLE Express Enzyme (Thermo Fisher Scientific) and inactivated the enzyme after 2 min by adding 0.5 mg/mL Soybean Trypsin Inhibitor (Thermo Fisher Scientific) to the TrypLE solution. We then resuspended the cells in 0.1% human recombinant Albumin (Sigma Aldrich) in DMEM-F/12 medium (Thermo Fisher Scientific) before centrifuging them at 150 × g for 3 min. To keep NES cells in a multipotent state, we resuspend them gently in NES medium, always aiming at keeping their density in the flasks between 30,000 and 250,000 cells/cm^2^ and splitting the content of each flask at 1:3 ratio at each passage. We prepared fresh NES medium weekly and replaced the medium in each flask daily. To differentiate NES towards NEU cells, we plated 78,000/cm^2^ NES cells resuspended in a differentiation medium composed of DMEM-F/12 medium (Thermo Fisher Scientific) supplemented with 1% N2 supplement (Thermo Fisher Scientific), 1% B27 supplement with Vitamin A (Thermo Fisher Scientific), and 1% Penicillin/Streptomycin (Thermo Fisher Scientific). Hereafter, we refer to this medium as differentiation (DIFF) medium. We prepared fresh DIFF medium every 2 weeks and replaced growth media every second day. As we no longer passaged the differentiating cell cultures, after day 10 we added 1 μg/mL Laminin (Sigma-Aldrich) to the DIFF medium to promote cell attachment to the flask. We monitored cell differentiation daily by using a phase contrast microscope (Axiocam, Zeiss) and performed immunofluorescence staining for various markers of neural cell differentiation, as described below.

### Immunofluorescence of NES cell differentiation markers

We cultured NES, NPC, and NEU cells on coated glass coverslips as described above. We rinsed the coverslips with DPBS Mg+/Ca+ at room temperature, followed by fixation of the cells in 1X PBS/4% formaldehyde (VWR) for 10 min at room temperature. We inactivated the unreacted formaldehyde with 1X PBS/125 mM glycine for 5 min at room temperature, followed by cell permeabilization with 1X PBS/0.2% Triton X-100 (Sigma-Aldrich) for 20 min at room temperature. We performed blocking in 1X PBS/3% bovine serum albumin (BSA) (Sigma-Aldrich) for 1 h at room temperature. We then incubated the samples with primary antibodies against stem cell or different neural lineage markers diluted in 1X PBS/3% BSA overnight at 4 °C (see Table 1 for a list of antibodies and dilutions used). The next day, we washed the coverslips three times with 1X PBS/0.1% Tween-20 (Sigma-Aldrich) (PBS-T) and then incubated them with the corresponding secondary antibody (Table 1) diluted in 1X PBS/3% BSA/0.02% Tween-20 for 1 hour at room temperature. We washed the samples twice with PBS-T, followed by incubation with 1X PBS/1 µg/mL Hoechst 33342 (Thermo Fischer Scientific) for 15 min at room temperature. We washed the samples twice in 1X PBS at room temperature, after which we mounted the coverslips in ProLong Gold Antifade Mountant (Thermo Fischer Scientific). We imaged the samples on a Nikon Ti2-E inverted microscope equipped with a ZYLA 4.2 sCMOS camera (Andor Technology) using a CFI Plan Fluor 40 × 0.75 Phase Microscope objective (Nikon). We acquired multiple image stacks per sample, each consisting of 41 focal planes spaced 0.2 µm apart.

**Table 1:** List of antibodies used for immunofluorescence.

Antibody	Source	Cat. no.	RRID*	Dilution
Anti-SOX-2	Millipore	AB5603	AB_2286686	1:500
Anti-Nestin	Millipore	MAB5326	AB_11211837	1:500
Anti-MAP2	Synaptic systems	188004	AB_2138181	1:1000
Anti-GFAP	Thermo Fisher Scientific	PA5-16291	AB_10980769	1:1000
Anti-53BP1	Novus	NB100–304	AB_10003037	1:1000
Alexa Fluor 488 Goat anti-Rabbit IgG (H + L)	Thermo Fisher Scientific	A-11008	AB_143165	1:500
Alexa Fluor 555 Donkey anti-Mouse IgG (H + L)	Thermo Fisher Scientific	A-31570	AB_2536180	1:500
Alexa Fluor 647 Goat anti-Guinea Pig IgG (H + L)	Thermo Fisher Scientific	A-21450	AB_2735091	1:500

^*^Identifier in the Research Resource IDentification (RRID) database (https://www.rrids.org/).

### Copy number profiling of NES and NEU cells

We prepared DNA sequencing libraries with the NEBNext Ultra II FD DNA Library Kit (New England Biolabs), using 400 and 200 ng of genomic DNA (gDNA) extracted from NES and NEU cells, respectively, as input. We enzymatically fragmented gDNA for 20 min aiming at obtaining fragments of size comprised between 150 and 350 base-pairs (bp). We performed a bead-based size selection of the libraries using SPRIselect beads (Beckman Coulter) with a 0.4 vol./vol. ratio for the first selection and 0.2 for the second selection. We indexed the libraries by performing 4 PCR cycles using indexes contained in the NEBNext Multiplex Oligos for Illumina Set 2 (New England Biolabs). We assessed the quality and size of the final libraries on a Bioanalyzer 2100 (Agilent Technologies). We sequenced the libraries on a NextSeq 500 machine (Illumina) using the NextSeq 500/550 High Output Kit v2 (Illumina), performing 75 sequencing cycles and 6 additional cycles for index sequencing. We pooled one library from NES and one from NEU cells into the same sequencing run, aiming at generating ~80 million reads per sample. After sequencing, we demultiplexed the raw reads using Illumina’s BaseSpace and aligned the demultiplexed reads to the human reference genome (GRCh37/hg19) with BWA-MEM (v0.7.17-r1188)^16^ using default options. Following this, we removed duplicate reads using the *MarkDuplicates* tool from the Genome Analysis ToolKit (v4.1.4.1)^17^. Lastly, we performed copy number calling based on circular binary segmentation^18^ using the R package QDNAseq^19^. with a genomic bin size of 100 kb and default options. To call amplifications and deletions we set a threshold of the Log2 ratio at 0.32 and −0.42, respectively.

### Preparation of cells for sBLISS

To obtain homogenous single-cell suspensions for sBLISS without damaging the cells, we first removed the culture medium and washed the cells with DPBS (Sigma Aldrich). For NEU cells, we added 3 mL of Accutase (Sigma Aldrich) pre-warmed at 37 °C onto the cells and incubated for 30 sec. After removing the Accutase, we added 2 mL of TrypLE Express Enzyme (Thermo Fisher Scientific) pre-warmed at 37 °C and incubated for 2 min at 37 °C. We then collected the dissociated cultures in 15 mL tubes, added 100 μL Neuron Isolation Enzyme (Pierce) to each sample, and incubated at 37 °C for 1 min. Afterwards, we added 2.5 mL of 0.5 mg/mL Soybean Trypsin Inhibitor (Thermo Fisher Scientific) to the TrypLE solution, and dissociated the cells by resuspending them thoroughly with 0.1% human recombinant Albumin (Sigma Aldrich) in 2.5 mL DMEM-F/12 medium (Thermo Fisher Scientific) before centrifuging them at 150 × g for 3 min. For NES and NPC cells, we dissociated the cells with 2 mL of TrypLE Express Enzyme pre-warmed at 37 °C and incubated for 2 min at 37 °C. We then collected the dissociated cultures in 15 mL tubes, added 2.5 mL per tube of 0.5 mg/mL Soybean Trypsin Inhibitor and dissociated the cells by resuspending them thoroughly after adding 2.5 mL DMEM-F/12 medium (Thermo Fisher Scientific) with 0.1% human recombinant Albumin (Sigma Aldrich) before centrifuging them at 150 × g for 3 min. We filtered single-cell NPC and NEU cell suspensions first through 100 μm and then through 40 μm cell strainers (Corning) to obtain debris-free cell suspensions. We fixed cells resuspended at a density of 10^6^ cells/mL in the appropriate growth medium by adding methanol-free paraformaldehyde (Thermo Fisher Scientific) to a final concentration of 2% and incubating the samples for 10 min at room temperature on a rolling shaker. Lastly, we inactivated the unreacted paraformaldehyde by adding glycine in 1X PBS to reach a final concentration of 125 mM and incubating for 5 min before washing the cells twice with DPBS. We stored samples prepared in this way at 4 °C for up to one week before proceeding with the sBLISS protocol (see below).

### In-suspension BLISS (sBLISS)

We performed sBLISS following the same protocol that we previously described in detail^11^. Briefly, we started by lysing the cell membrane in a lysis buffer containing 10 mM Tris-HCl/10 mM NaCl/1 mM EDTA/0.2% Triton X-100 pH 8 and incubated the samples for 60 min on ice. We then pelleted the cells at 150 × g for 5 min at room temperature, removed the supernatant, resuspended them in a permeabilization buffer consisting of 10 mM Tris-HCl/150 mM NaCl/1 mM EDTA/0.3% SDS pH 8 pre-warmed at 37 °C, and incubated the samples for 60 min at 37 °C. Next, we pelleted the cells at 150 × g for 5 min at room temperature, washed them twice with CutSmart Buffer (New England Biolabs)/0.1% Triton X-100 pre-warmed at 37 °C. To convert DSBs containing overhangs into a form that can be ligated to blunt adapters, we used the Quick Blunting Kit (New England Biolabs) and resuspended the cells in a final blunting reaction volume of 100 μL, followed by 60 min incubation at room temperature. We then washed the cells with CutSmart Buffer/0.1% Triton X-100 at room temperature and proceeded to *in situ* ligation of sBLISS adapters to the blunted DSB ends. For ligation we used 25 Weiss units (U) of 5 U/μL T4 DNA Ligase (ThermoFisher Scientific) and incubated the cells at 16 °C for 20–24 hours in a 100 μL ligation reaction mix containing 3 μL of 50 mg/mL BSA (ThermoFisher Scientific) and 12 μL of 10 mM ATP (ThermoFisher Scientific). For a sample containing ~10^6^ cells, we used 4 μL of sBLISS adapter at 10 μM prepared as we described before^11^. The complete list of the sBLISS adapters used to generate the datasets described here is available in Supplementary Table 2. After overnight ligation, we washed the cells twice with CutSmart Buffer/0.1% Triton X-100 at room temperature, and then extracted gDNA by resuspending the cells in 100 μL DNA extraction buffer containing 10 mM Tris-HCl/100 mM NaCl/50 mM EDTA/1% SDS pH 7.5 and adding 10 μL per sample of 800 U/mL Proteinase K (New England Biolabs). We incubated the samples for 14–18 h at 55 °C in a thermal mixer set at 800 shakes-per-minute, followed by addition of 10 μL of fresh Proteinase K the next morning and continuing the incubation for 1 hour. We then heat-inactivated the Proteinase K for 10 min at 95 °C and extracted gDNA using a 25/24/1 (vol./vol./vol.) solution of Phenol-Chloroform-Isoamyl Alcohol with 10 mM Tris pH 8.0, 1 mM EDTA (Sigma-Aldrich) and Chloroform (Merck), followed by ethanol precipitation. After drying, we dissolved the DNA pellets in 102 μL of Tris-EDTA by placing the tubes in a thermal mixer set to 50 °C for 15 min while shaking at 1,100 shakes-per-minute. After cooling the samples to 4 °C, we sonicated 100 μL of each sample using BioRuptor Plus (Diagenode) with the following settings: 30 sec on, 60 sec off, high intensity, 30 cycles. We concentrated the sonicated gDNA samples using AMPure XP beads (Beckman Coulter) and assessed the fragment size using a BioAnalyzer 2100 (Agilent Technologies). We aimed at obtaining gDNA fragments with sizes ranging from 300 to 800 bp, with a peak around 400–600 bp. We stored the sonicated and purified gDNA samples at –20 °C until we performed *in vitro* transcription (IVT) and library preparation. For IVT, we used 90–300 ng of each sonicated gDNA sample (see Supplementary Table 1 for the exact amount used for each sample) and the MEGAscript T7 Transcription Kit (Thermo Fisher Scientific) following the manufacturer’s instructions, with the exception that we carried out all reactions for 14 hours at 37 °C after adding RiboSafe RNAse Inhibitor (Bioline) to each sample. Upon IVT completion, we degraded the gDNA by adding 2 U of RNase-free DNAse I (Thermo Fisher Scientific) to each sample and purified the amplified RNA (aRNA) with RNAClean XP beads (Beckman Coulter). Next, we ligated each aRNA sample to the Illumina RA3 adapter (purchased from Integrated DNA Technologies) using T4 RNA Ligase 2 (New England Biolabs) and incubated the samples for 2 hours at 25 °C. Thereafter, we reverse transcribed the ligated aRNA using the Illumina RTP primer (purchased from Integrated DNA Technologies) and SuperScript IV Reverse Transcriptase (Thermo Fisher Scientific), by following the manufacturer’s instructions except for the incubation time, which we extended to 50 min at 50 °C followed by 10 min heat-inactivation at 80 °C. To prevent RNAse activity, we added RNaseOUT (Thermo Fisher Scientific) during the RA3 adapter ligation and during the reverse transcription. We amplified the resulting libraries using NEBNext Ultra II Q5 Master Mix (New England Biolabs), the Illumina RP1 primer (purchased from Integrated DNA Technologies), and one of the Illumina RPIX index primers (purchased from Integrated DNA Technologies), by performing 8 PCR cycles in 400 μL split into 8 PCR tubes to increase library complexity. The sequences of the RT, RP1 and RPIX primers used to generate the datasets described here are available in Supplementary Table 2. After PCR, we purified and size-selected the libraries using two-sided AMPure XP bead (Beckman Coulter) purification, aiming at obtaining libraries with a fragment size ranging between 300 and 900 bp. We assessed the final libraries on a BioAnalyzer High Sensitivity DNA chip (Agilent Technologies) and quantified them using the Qubit dsDNA HS Assay kit (Thermo Fisher Scientific). We sequenced all the libraries on a NextSeq 500 machine (Illumina) using the NextSeq 500/550 High Output Kit v2 (Illumina), performing 75 sequencing cycles and additional 6 cycles for index sequencing. We sequenced multiple indexed sBLISS libraries together aiming at obtaining ~25 million reads per library.

After sequencing, we demultiplexed the raw data based on index sequences using Illumina’s BaseSpace and retrieved the corresponding FASTQ files. We processed the raw sequencing reads using the sBLISS pipeline that we previously described in detail^11^. Briefly, the pipeline first selects reads containing the expected sBLISS prefix comprised of the unique molecular identifier (UMI; 8 nucleotides, nt) and the sample barcode (BC; 8 nt) using SAMtools^20^ (v1.10) and Scan for Matches^21^, allowing at most one mismatch in the barcode. The pipeline then clips the prefixes off and stores them, while the trimmed reads are aligned to the human reference genome (GRCh37/hg19) using BWA-MEM^22^ (v0.7.17-r1188) with default options. Afterwards, the pipeline discards the reads with mapping quality scores lower than 30 and removes PCR duplicates by searching for adjacently mapped reads (at most 10 bp apart along the reference genome) with at most one mismatch in their UMI sequence. Finally, the pipeline returns a list of unique DSB locations and the corresponding number of unique UMIs identified at each position, which we used for all downstream analyses described below. To visualize the distribution of DSBs along selected genes, we uploaded the corresponding BED files to the UCSC Genome Browser (https://genome.ucsc.edu/cgi-bin/hgTracks) and selected the *squish* display mode in the Track Settings.

### Quantification of DSBs by immunofluorescence

We performed immunofluorescence staining for the DSB marker 53 binding protein 1 (53BP1) following the same procedure described above for stem and neural differentiation markers, except for the imaging part (see Table 1 for a list of antibodies and dilutions used). For imaging, we mounted the coverslips in non-hardening Vectashield medium (Vector labs). We imaged the samples on the same Nikon Ti-E microscope equipped with a Sona 4.2B-6 sCMOS camera (Andor Technology) using a using a CFI Plan Apochromat Lambda 100X Oil objective (Nikon). We acquired 10 image stacks per sample, each consisting of at least 48 focal planes spaced 0.2 µm apart, aiming at imaging at least 150 cells for each differentiation stage (NES, NPC, and NEU). To segment the cells, we first used a custom script written in MATLAB (see Code Availability) to generate 2D nuclei segmentation masks using the Otsu’s method to find a global threshold of the fluorescence intensity in the DNA channel for each field of view. We visually inspected and, if needed, manually corrected all the segmentation masks. We then measured the fluorescence intensity in the channel corresponding to 53BP1 by integrating the images axially over the segmentation masks, using the mid 48 slices in each z-stack. When the images contained more than 48 slices, we selected a range of z-values where the corresponding gradient magnitude was as high as possible (this corresponds to performing an axial centering around the middle section of the nuclei).

### Total RNA-Seq

We cultured NES, NPC, and NEU cells as described above and rinsed them with DPBS Mg+/Ca+ (Sigma-Aldrich). We added 1 mL of TRI Reagent Solution (Thermo Fisher Scientific) per million cells directly to the cells attached to the flasks. After incubating for 5 min at room temperature, we detached the cells by pipetting the TRI Reagent Solution on them multiple times and pipetting the detached cells up-and-down multiple times until the solution became clear. We then transferred the solution to a 1.5 mL tube and stored the samples at −20 °C overnight. The next day, we added 20% (vol./vol.) chloroform (Sigma-Aldrich) to each sample, mixed well and incubated for 3 min at room temperature, followed by centrifugation at 12,500 × g for 15 min at 4 °C. We then transferred the upper phase and thoroughly mixed this with an equal volume of ice-cold 70% ethanol. Next, we pipetted the mixture onto a PureLink RNA Mini Spin Cartridge (Thermo Fisher Scientific) and proceed to RNA purification following to the manufacturer’s instructions. RNA-Seq libraries were prepared by the National Genomics Infrastructure at the Science for Life Laboratory (SciLifeLab) in Stockholm, Sweden, using the TruSeq Stranded Total RNA Library Prep kit with RiboZero from Illumina. We assessed and quantified the final libraries on a BioAnalyzer 2100 (Agilent Technologies) and Qubit using a High Sensitivity RNA chip (Thermo Fisher Scientific). We sequenced the libraries on a NextSeq 500 machine (Illumina) using the NextSeq 500/550 High Output Kit v2 (Illumina) and performing 75 sequencing cycles and 6 additional cycles for index sequencing. We pooled three replicates for each of the three cell types, aiming at obtaining at least 50 million reads per library.

After sequencing, we demultiplexed the raw data based on the index sequences using Illumina’s BaseSpace and downloaded the corresponding FASTQ files. We performed sequencing quality checks for all the experiments using FastQC (https://www.bioinformatics.babraham.ac.uk/projects/fastqc/, v0.11.9). We removed the adapter sequences using TrimGalore^23^ (v0.6.4_dev) with default parameters. We filtered the reads against human rRNA and tRNA sequences obtained from NCBI using Bowtie2^24^ (v2.4.1) with the option --*sensitive-local*. We used the reads that failed to align in the previous step as input for STAR^25^ (v2.7.0e) and mapped them to the human reference genome (GRCh37/hg19) using the GENCODE^26^ (v19) gene reference annotation with the following parameters: --*twopassMode Basic* --*alignSJoverhangMin 8* --*alignSJDBoverhangMin 1* --*sjdbScore 1* --*outFilterMultimapNmax 1* --*outFilterType BySJout* --*outFilterMismatchNmax 999* --*outFilterMismatchNoverReadLmax 0.04* --*outSAMattributes All* --*outSAMtype BAM SortedByCoordinate* --*alignIntronMin 20* --*alignIntronMax 1000000* --*alignMatesGapMax 1000000*. We removed PCR duplicates using the Picard MarkDuplicates tool (http://broadinstitute.github.io/picard/, v2.21.7) with default parameters. We carried out transcript expression quantification using Salmon^27^ (v1.2.0) in pseudo-alignment mode with library type (--*l ISR*) and correction of sequence-specific (--*seqBias*) and position-specific (-*posBias*) biases, and the GENCODE (v19) annotation and corresponding transcriptome as references. We imported the transcripts per million (TPM) tables obtained with Salmon into R (https://www.r-project.org/, v4.0.3) using the tximport package^28^ with *lengthScaledTPM* scaling method and no scaling for differential gene expression analysis. We performed gene expression levels normalization across samples and differential gene expression calculations using the DESeq 2 R package and the IHW R package for Independent Hypothesis Weighting. We adjusted the *P* values for multiple hypotheses testing with the Benjamini and Hochberg method^29^ and used a false discovery rate (FDR) of 0.001 and absolute Log2 fold-change of 1 as thresholds to extract genes significantly changing their expression between conditions. We applied a variance stabilizing transformation (VST) using the *vst* function from the DESeq 2 package. We then extracted the expression values of differentially expressed genes, calculated their z-score across conditions and replicates and the Euclidean distance between gene vectors using the *dist* function from the stats R package, and performed hierarchical clustering using the *hclust* function from the stats R package. The number (5) of final clusters was obtained through the *cutree* function from the R Stats package with h parameter set to 5. We performed all Gene Ontology (GO) enrichment analyses using the Biological Processes (BP) ontology and the *compareCluster* function from the clusterProfiler R package. We carried out the individual GO terms (*e.g*., Neurogenesis, Axonogenesis, *etc*.) enrichments across differentially expressed gene clusters by generating the corresponding contingency tables and applying the Fisher’s exact test to assess their statistical significance using the *fisher .test* function from the Stats R package, with alternative hypothesis set to *greater*. We retrieved the individual GO terms by performing manual queries across the AmiGO 2 database (http://amigo.geneontology.org/).

### Hi-C

We prepared Hi-C libraries using the Arima-HiC kit (Arima Genomics) following the manufacturer’s instructions. Briefly, we crosslinked the cells with 2% formaldehyde (following the same procedure described above for preparing cells for sBLISS) and then used approximately 1 million fixed cells as input for each cell type. Subsequently, we used 2.5 μg (for NES) or 1.5 μg (for NPC and NEU) of Hi-C template for biotin pull-down and library preparation according to the Arima Genomics User Guide for Library Preparation using KAPA Hyper Prep Kit. Specifically, we used 6 (for NES) or 8 (for NPC and NEU) PCR cycles for library amplification. We then pooled corresponding Arima-HiC replicate libraries and sequenced each pool on one SP flowcell on the NovaSeq 6000 system (Illumina) in paired-end sequencing mode aiming at obtaining ~800 million reads per library. Sequencing was carried out at the National Genomics Infrastructure at the Science for Life Laboratory (SciLifeLab) in Stockholm, Sweden.

After sequencing, we processed the raw sequencing data using HiCUP^30^ (v0.7.4) with default parameters. Briefly, the pipeline employs Bowtie2^24^ (v2.4.1) to align the reads to the human reference genome (GRCh37/hg19) and filters out experimental artefacts (*i.e*., circularized, re-ligated, and duplicate reads). We generated ‘digest’ files using the *hicup_digester* command with the option --*arima*. We used the HiCUP output files (BAM format) containing only valid, non-redundant read pairs, as input for pairtools (https://pairtools.readthedocs.io/en/latest/, v0.3.0). First, we converted and sorted the BAM files into pairsam format using the *parse* and *sort* modules in pairtools. In addition to individual replicates, we generated pooled samples for each cell type (NES, NPC, and NEU) by merging matched replicates using the *merge* module in pairtools. We marked read duplicates using the pairtools *dedup* module with option *–mark-dups* and filtered the results selecting only specific pair types (*i.e*., ‘UU’, ‘UR’ and ‘RU’) via the pairtools *select* module, which produced an output in pairs format. Finally, we added the fragment information using the *fragment_4dnpairs.pl* convenience script provided alongside the Juicer pipeline^31^, and converted the pairs files into hic format using the *Pre* module from Juicer-Tools (v1.22.01). Unless explicitly required by the software/package used, we normalized all contact matrices with Knight-Ruiz Matrix Balancing (KR) using Juicer-Tools. For each chromosome, we extracted from the hic files the intra-chromosomal interactions matrix at 1 megabase (Mb) and 100 kb resolution using Straw^31^ without normalization. We assessed the reproducibility of Hi-C data using the GenomeDISCO^32^ (v1.0.0) *run_all* module.

### A/B compartments calling

For each cell type (NES, NPC, and NEU), we extracted the first and second eigenvectors at 1 Mb resolution from the hic files, using the *compartments* module from the FAN-C^33^ toolkit (v0.9.17). For each chromosome, we determined the correctness of the eigenvectors by correlating the first and second eigenvector of each developmental stage with the first eigenvector of the others and switched the first with the second eigenvector if the Pearson’s correlation of the latter was higher than the former. In addition, although FAN-C orients the eigenvectors by using the average GC-content information, we re-assigned the orientation of each eigenvector by using the average RNA-Seq signal across positive and negative regions. We defined regions with high RNA-Seq signal as ‘A’ compartments and regions with low RNA-Seq signal as ‘B’ compartments.

### TAD boundaries calling and classification

To call TAD boundaries, we used Straw^34^ without normalization to extract the intra-chromosomal interaction matrix for each chromosome at 100 kb resolution. We then imported the hic files into R and used them as input for the *TimeCompare* function in TADCompare^35^. We classified TAD boundaries into six different categories: (1) Early Appearing (*i.e*., boundaries detected in NPC and NEU but not in NES); (2) Early Disappearing (*i.e*., boundaries detected in NES but not in NPC and NEU); (3) Late Appearing (*i.e*., boundaries detected in NEU but not in NES and NPC); (4) Late Disappearing (*i.e*., boundaries detected in NES and NPC but not in NEU); (5) Dynamic (*i.e*., boundaries detected only in NES and NEU or only in NPC); and (6) Common (*i.e*., boundaries detected in all three cell types). In addition, for each cell line we extracted the insulation scores and plotted their average across a 200 kb region centered on the TAD boundary, using the *insulation* and *boundaries* modules from the FAN-C toolkit.

### Chromatin loops calling and classification

To call chromatin loops at 5 kb resolution, we applied Mustache^36^ (v0.1.4) to the hic files using the --*pThreshold 0.1* parameter. Then, we imported the list of chromatin loops into R and classified them in six categories, as described above for TAD boundaries: (1) Early Appearing (*i.e*., loops absent in NES and present in NPC and NEU); (2) Early Disappearing (*i.e*., loops detected in NES but not in NPC and NEU); (3) Late Appearing (*i.e*., loops detected in NEU but not in NES and NPC); (4) Late Disappearing (*i.e*., loops detected in NES and NPC but not in NEU); (5) Dynamic (*i.e*., loops detected in NES and NEU or only in NPC); and (6) Common (*i.e*., loops detected in all three cell types).

### Analysis of DSBs at promoter regions and along gene bodies

We first selected all expressed protein-coding genes (average TPM ≥ 1 across all RNA-Seq samples) and computed the number of DSBs in their promoter (defined as the region from 2 kb upstream to 1 kb downstream of the TSS) or in their gene body (*i.e*., from the TSS to the transcription end site or TES). The only exception was when we calculated the ratio of DSBs in the promoter over the gene body, in which case we computed the number of DSBs from 1 kb downstream of the TSS to the TES. We discarded genes shorter than 2 kb to avoid overestimating the signal along the gene body for regions shorter than 1 kb. To compare the DSB burden across promoter regions and gene bodies within the same sample, we normalized the DSB counts per million mapped reads (CPM) or per kilobase gene length per million mapped reads (CPKM), respectively. To compare the DSB burden across different samples (*e.g*., to compare DSB metaprofiles around the TSS in NES, NPC, and NEU cells), we downsampled the DSB counts in each sample to the size of the smallest dataset (~15 million DSBs). We added a pseudo-count for plotting or to avoid null denominators in ratios whenever we applied a logarithmic transformation.

### Analysis of DSBs at TAD boundaries and around chromatin loop anchors

To compute the DSB burden around TAD boundaries, we used a window of 200 kb centered on each TAD boundary to calculate how many of the downsampled DSBs (~15 millions) were mapped in these regions. To assess DSBs around chromatin loop anchors, we generated a list of significant chromatin loops at 5 kb resolution for each differentiation stage using *HiCCUPS* from Juicer tools (v1.22.01). We used the central positions of the upstream and downstream loop anchors as loop start and end reference points, respectively. We then used a window of 40 kb centered on these reference points to calculate how many of the downsampled DSBs (~15 millions) were mapped in these regions. Lastly, we normalized the generated metadata profiles by dividing the DSB counts by the lowest value of the density function, which was then considered as signal baseline.

### Analysis of DSBs in NDD risk genes

We manually compiled a list of genes associated with increased risk for either SCZ or ASD by parsing the Supplementary Data in ref. ^37^ and Table S2 in ref. ^38^, respectively, and retaining only unique gene names (see Supplementary Table 3). We then mapped the gene names to their corresponding Entrez identifier using the AnnotationDbi^39^ R package. We computed the number of DSBs in the promoter region and along the gene body of each gene as described above and used the distribution of all protein-coding genes (after filtering out those included in the SCZ and ASD risk genes list) as background reference.

## Data records

sBLISS (Datasets 1–6)^13^, RNA-Seq (Datasets 7–15)^13^, and Hi-C (Datasets 16–21)^13^ FASTQ files have been deposited in the Sequence Read Archive (SRA) and are publicly accessible through the following link: https://www.ncbi.nlm.nih.gov/bioproject/PRJNA798046. sBLISS BED files are available at: 10.6084/m9.figshare.18530531.v2^14^. Immunofluorescence raw images and 2D nuclear segmentation masks can be accessed through the following link: 10.17044/scilifelab.19630374^40^.

## Technical validation

### Validation of the 2D cell culture model system of neural differentiation

We first aimed at validating our *in vitro* model of neural cell fate specification by performing immunofluorescence staining for various neural lineage markers (Methods). As expected, NES cells expressed neuroepithelial stem cell protein (Nestin) and sex determining region Y-box 2 (SOX2), two markers of actively self-renewing neuroepithelial stem cells, and showed neural rosette formation (Fig. 1b, NEU). After 6 days of *in vitro* differentiation, the cells started to divide less frequently and short projections expressing the neuronal marker microtubule-associated protein 2 (MAP2) began to form (Fig. 1b, NPC). After 35 days of differentiation, the cultures consisted mainly of neuronal cells with highly entangled MAP2-positive fibers and, amidst them, few cells expressing the glial fibrillary acidic protein 2 (GFAP) marker of differentiation towards astrocytes (Fig. 1b, NEU).

It has been reported that reprogramming of somatic cells and prolonged passaging of human iPSCs can result in the formation of karyotypic abnormalities such as chromosomal amplifications or deletions^41^. The iPSC-derived NES cell line that we used to generate the datasets described here (see Methods) was previously shown to be karyotypically normal and stable over multiple passages^15,42^. However, to exclude that chromosomal alterations formed during the course of our experiments, we performed low-pass (1X sequencing depth) whole-genome sequencing followed by copy number calling using genomic DNA extracted from NES cells and NEU cells after 35 days of differentiation (Methods). For both differentiation stages, the genome-wide copy number profiles were flat (Fig. 1c,d), indicating that the cells remained karyotypically stable throughout our experiments.

To further characterize our model system, we performed three total RNA-Seq experiments on NES, NPC, and NEU cells (from different NES passages), followed by differential gene expression and gene ontology (GO) analysis (Methods). Experimental replicates corresponding to the same differentiation stage clustered together, whereas the largest difference was observed between NES and NEU samples, as expected (Fig. 1e). We identified 4,583 differentially expressed genes forming two larger and three smaller clusters marked by different expression patterns (Fig. 1f). Cluster 1 was significantly enriched in genes linked to DNA replication, which became downregulated during the transition from NES to NEU, as expected given that the latter cells stop cycling (Fig. 1f,g). On the other hand, cluster 2 and, to a lesser extent, clusters 3–5 were significantly enriched in GO terms related to differentiation towards neural lineages and neuronal functions, such as ‘Neurogenesis’, ‘Neuron differentiation’, and ‘Axonogenesis’ (Fig. 1h–n). Altogether, these results demonstrate that our *in vitro* differentiation system is a good proxy of the morphological and transcriptional changes that occur during neural cell lineage specification.

### sBLISS reproducibly detects endogenous DSBs

**Fig. 2:**
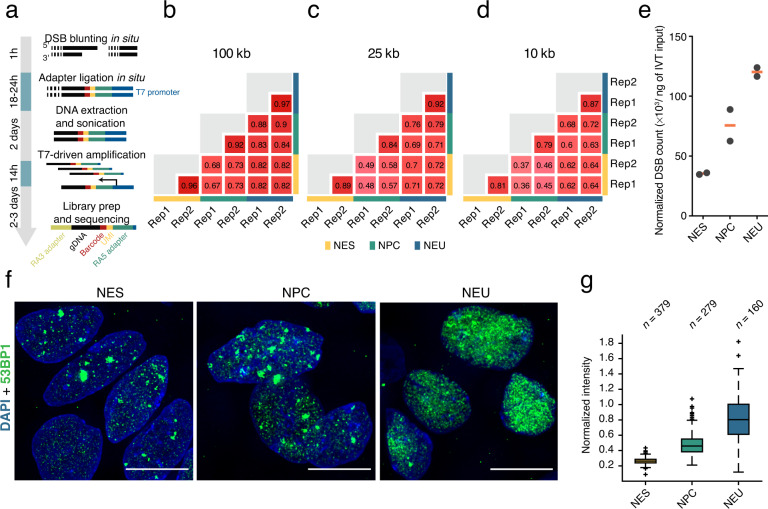
Overview and validation of sBLISS. (**a**) sBLISS workflow and schematic representation of the adapters used to tag individual DSB ends and to amplify the genomic DNA (gDNA) sequence downstream by *in vitro* transcription. UMI, unique molecular identifier. T7, T7 phage RNA polymerase. RA3/5, Illumina adapters. (**b**–**d**) Reproducibility of DSB counts at different genomic resolutions between two sBLISS replicate (Rep) experiments in NES, NPC, and NEU cells. The numbers in the red squares represent the Pearson’s correlation coefficient. (**e**) Normalized counts of DSB ends detected by sBLISS in each of the six sBLISS datasets described here. The DSB counts were normalized to the amount (in ng) of genomic DNA used as input in the *in vitro* transcription (IVT) step in sBLISS. Each grey dot represents one replicate experiment. Orange bars, mean value. (**f**) Maximum z-projections of wide-field epifluorescence microscopy z-stacks showing the expression of the DSB marker 53BP1 in NES, NPC, and NEU cells. Representative fields of view are shown. Scale bars, 50 μm. Blue, DNA staining with Hoechst 33342. (**g**) Normalized 53BP1 nuclear intensity in the images of which those shown in (**f**) are representative examples. For each segmented nucleus, we normalized the intensity in the fluorescence channel of the 53BP1 antibody to the intensity of the DNA staining channel (see Methods). Each boxplot extends from the 25th to the 75th percentile, the horizontal bars represent the median, and whiskers extend from –1.5 × IQR to + 1.5 × IQR from the closest quartile, where IQR is the inter-quartile range. Black dots, outliers.

Next, we performed two sBLISS experiments on NES, NPC, and NEU cells (from different NES passages), following the same procedure that we previously described in detail^11^ (Fig. 2a and Methods). We first compared DSB counts across replicates at decreasing genomic bin sizes (100, 25, and 10 kb). DSB counts were highly correlated between replicates (Pearson’s correlation coefficient (PCC) between replicates: 0.95 ± 0.02, 0.88 ± 0.03, 0.82 ± 0.03 at 100, 25, and 10 kb resolution, respectively, mean ± s.d.), highlighting the reproducibility of sBLISS (Fig. 2b–d). To compare the DSB counts between differentiation stages, we normalized the DSB counts based on the genomic DNA input to the IVT step in sBLISS (Methods). The normalized DSB burden progressively increased as cells differentiated, with NEU samples yielding the highest number of DSB ends sequenced (Fig. 2e). To validate this finding using an orthogonal approach, we performed immunofluorescence staining for the DSB marker, 53BP1 (Methods). Indeed, NPC and especially NEU cells displayed substantially higher 53BP1 levels compared to NES cells (Fig. 2f,g). Altogether, these results demonstrate that sBLISS is a valid approach to reproducibly capture changes in the burden of endogenous DSBs.

### DSBs are enriched at the promoter and along the gene body of highly transcribed genes

**Fig. 3:**
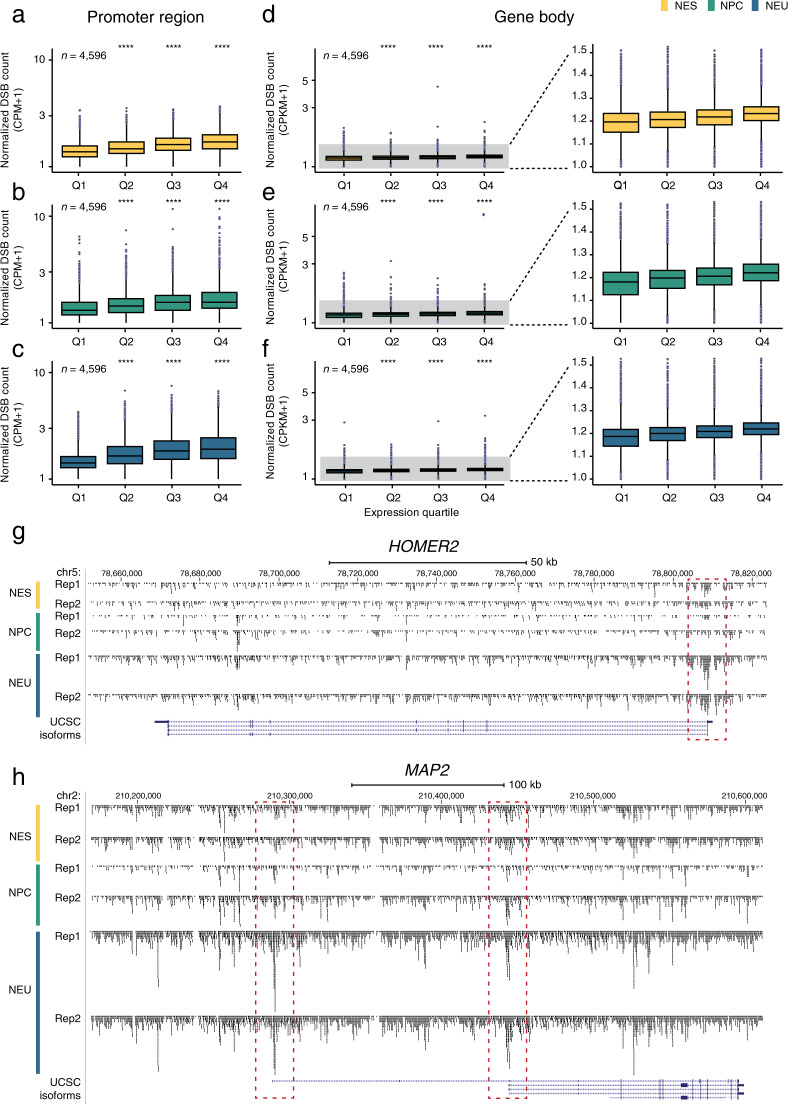
Endogenous DSBs are enriched in the promoter region and along the gene body of highly expressed protein-coding genes. (**a**–**c**) Distributions of normalized DSB counts in a 3 kb window (from 2 kb upstream to 1 kb downstream) around the transcription start sites (TSS) of human protein-coding genes classified in four different quartiles (Q) based on their expression levels determined by RNA-Seq. CPM, DSB count per million reads calculated as number of DSBs divided by number of reads times one million. *n*, number of genes in each expression quartile. Asterisks indicate a *P* value lower than 0.0001 (Wilcoxon test, two-tailed) comparing the distribution below them with the Q1 distribution in the same plot. (**d**–**f**) Same as in (**a–c**), but for DSBs along the gene body of human protein-coding genes (from the first TSS to the last transcription end site of each gene). The part of the boxplots highlighted in grey is magnified on the right. (**g,h**) Visualization of mapped DSBs along two genes using the *squish* option in the UCSC genome browser. The dashed red rectangles indicate the enrichment of DSBs around the TSSs of the two genes. In all the boxplots shown in the figure, each boxplot extends from the 25th to the 75th percentile, the horizontal bar represents the median, and whiskers extend from –1.5 × IQR to + 1.5 × IQR from the closest quartile, where IQR is the inter-quartile range. Black dots, outliers.

Using sBLISS, we previously showed that endogenous DSBs are enriched around the TSS of transcribed protein-coding genes in immortalized chronic myeloid leukemia TK6 cells, as well as in primary mouse enterocytes^11^. Other studies using alternative DSB detection approaches, such as DSBCapture^43^, END-seq^44^. and High-Throughput Genome-wide Translocation Sequencing (HTGTS)^45^, have also reported similar enrichment patterns of both endogenous and treatment-induced DSBs around the TSS of actively transcribed genes in different cell types, including mouse neural stem cells^4,6–8,11,43,46,47^. Hence, to further validate our sBLISS datasets, we assessed whether the endogenous DSBs detected by sBLISS in NES, NPC, and NEU cells are also enriched around the TSS of protein-coding genes in a transcription-associated manner (Methods). The normalized DSB counts around the TSS were significantly higher (*P* < 0.0001, Wilcoxon test, two-tailed) in the top expression quartile gene group, in all three differentiation stages analyzed (Fig. 3a–c). The DSB burden around the TSS of highly expressed genes was significantly higher (*P* < 0.0001, Wilcoxon test, two-tailed) in NEU compared to NES cells, suggesting that differentiated, post-mitotic neuronal cells might experience higher levels of transcription-related torsional stress in promoter regions. Next, we investigated whether the gene body of highly expressed genes also harbors more DSBs compared to lower expressed genes. Indeed, in all three differentiation stages, protein-coding genes in the top expression quartile displayed significantly higher (*P* < 0.0001, Wilcoxon test, two-tailed) normalized DSB counts along the gene body compared to genes in the bottom quartile (Fig. 3d–f). Visual inspection of the DSB tracks along selected genes confirmed that DSBs accumulate around the TSS and along the gene body of genes upregulated during the differentiation of NES cells towards NEU (Fig. 3g,h). Altogether, these results demonstrate that highly expressed genes at different stages of neural cell fate specification are hotspots of endogenous DSB formation, in line with previous observations by our and other groups^4,6–8,11,43,46,47^, further corroborating the validity of our datasets.

### CpG content at promoters correlates with fragility

**Fig. 4:**
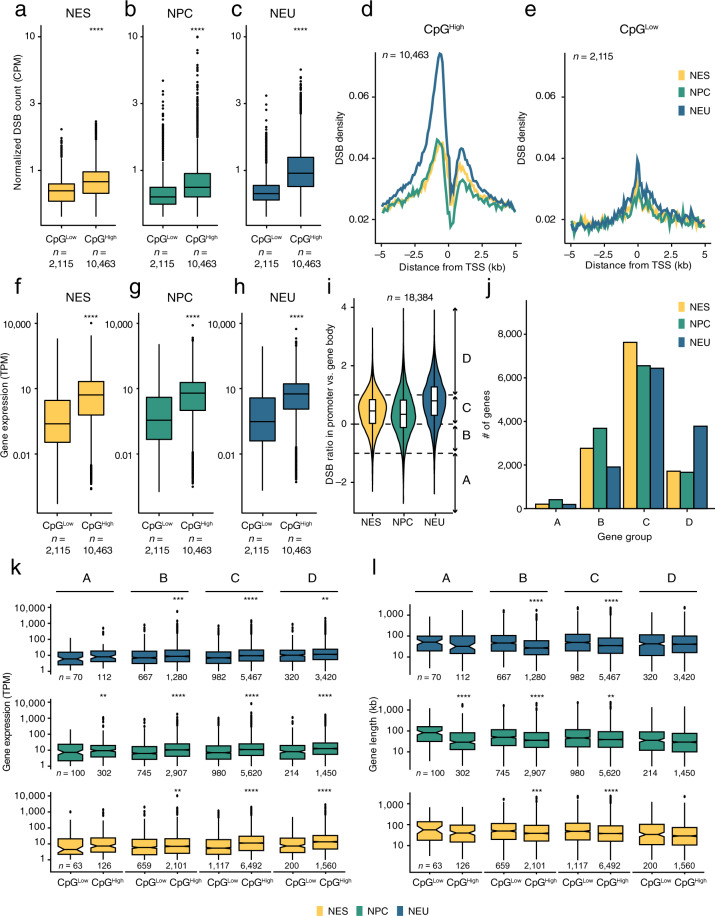
CpG-rich promoters are highly fragile. (**a**–**c**) Distributions of normalized DSB counts in a 3 kb window (from 2 kb upstream to 1 kb downstream) around the transcription start sites (TSS) of human protein-coding genes, for genes with high (CpG^High^) or low (CpG^Low^) levels of CpG dinucleotides in their promoter region. CPM, DSB count per million reads calculated as number of DSBs divided by number of reads times one million. *n*, number of genes in each group. (**d,e**) Metaprofiles of the DSB density around the TSS of human protein-coding genes classified as CpG^High^ (d) or CpG^Low^ (**e**) based on the frequency of CpG dinucleotides in their promoter region. *n*, number of genes. (**f**–**h**) Same as in (**a**–**c**) but for gene expression levels. TPM, transcripts per million. (**i**) Distributions of the ratio between the number of DSBs in the promoter (from 2 kb upstream to 1 kb downstream of the TSS) and along the gene body (from the first TSS of the gene to the last transcription end site), for all human protein-coding genes (*n*) in NES, NPC, and NEU cells. Each distribution was arbitrarily divided into four regions as following: A (–Inf; –1]; B (–1; 0]; C (0; 1]; D (1; Inf]. The violin plots extend from minimum to maximum, and the boxplots inside the violins extend from the 25th to the 75th percentile, with the horizontal bar representing the median, and whiskers extending from –1.5 × IQR to + 1.5 × IQR from the closest quartile, where IQR is the inter-quartile range. (**j**) Number of genes in each of the four groups shown in (**i**). (**k**) Distributions of gene expression levels measured by RNA-Seq in the four gene groups shown in (**i**), for genes classified as CpG^High^ or CpG^Low^ based on the frequency of CpG dinucleotides in their promoter region. The number of genes (*n*) in each group is shown below each boxplot. (**l**) Same as in (**k**) but for gene length in kiloSbases (kb). Gene length was calculated as the distance from the TSS to the transcription end site of each gene. In all the boxplots shown in the figure, each boxplot extends from the 25th to the 75th percentile, the horizontal bar represents the median, and whiskers extend from –1.5 × IQR to + 1.5 × IQR from the closest quartile, where IQR is the inter-quartile range. Black dots, outliers. The asterisks in (**a**–**c**), (**f**–**h**) and (**k**,**m**) indicate a *P* value less than 0.01 (**), 0.001 (***) or 0.0001 (****) (Wilcoxon test, two-tailed). ns, not significant.

To further investigate endogenous DSBs at promoter regions, we assessed whether the CpG content of the sequence surrounding the TSS is associated with the frequency of DSBs detected by sBLISS. 72% of human promoter sequences contain CpG islands that are considered important for regulation of development and that have been associated with constitutively nucleosome-depleted regions, altered modifications and positioning of nucleosomes, and distinct patterns of transcription initiation^48,49^. We therefore examined the DSB distribution at protein-coding gene promoters classified as CpG^High^ or CpG^Low^ based on their CpG content (Methods). In all three stages of differentiation, CpG^High^ promoters carried a significantly higher (*P* < 0.0001, Wilcoxon test, two-tailed) burden of DSBs compared to CpG^Low^ ones (Fig. 4a–c). Notably, CpG^High^ promoters in NEU cells showed the highest density of endogenous DSBs in the region upstream of the TSS (Fig. 4d,e). Comparison with RNA-Seq data showed that genes with a CpG^High^ promoter were expressed at significantly higher levels in all three stages of differentiation, suggesting that the CpG content might indirectly affect the fragility of promoters by mediating high transcriptional activity in these regions (Fig. 4f–h).

Next, we wondered how DSBs are distributed along the promoter and gene body of the same gene. To this end, we computed the ratio between the normalized DSB counts in the promoter region and along the gene body for all human protein-coding genes and classified the genes into four different groups (A, B, C, D) based on the calculated ratio (Fig. 4i). Interestingly, considerably more genes were assigned to group D (*i.e*., genes with DSBs predominantly concentrated in the promoter region) in NEU compared to NES and NPC cells, whereas more genes were assigned to group B (*i.e*., genes with DSBs more abundant in the gene body compared to the promoter region) in NES and NPC compared to NEU cells (Fig. 4j). Expression levels progressively increased from group A to group D and were consistently higher for genes with CpG^High^ promoters (Fig. 4k). The latter genes were, on average, also shorter compared to genes harboring CpG^Low^ promoters (Fig. 4l). Altogether, these results indicate that gene fragility is generally higher in NEU compared to NES cells, and that the frequency and pattern of DSBs along protein-coding genes correlate with multiple factors, including CpG abundance in the promoter region, gene length, and expression levels.

### DSBs are enriched in the A chromatin compartment

**Fig. 5:**
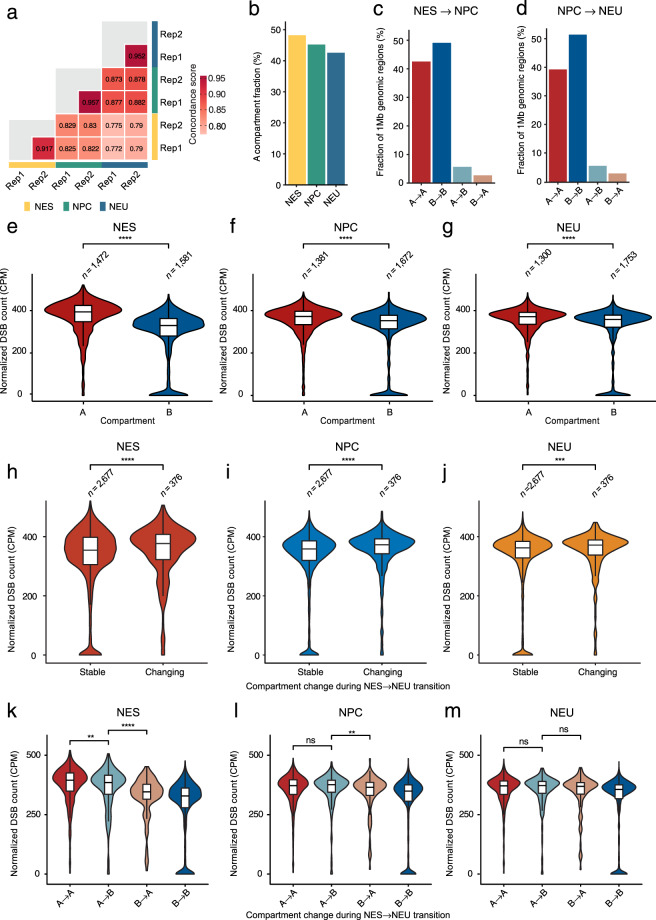
Endogenous DSBs are enriched in the active (A) chromatin compartment. (**a**) Concordance matrix revealing high similarity between the Hi-C replicates generated from NES, NPC, and NEU cells (100 kb resolution). (**b**) Fraction of the genome (1 Mb resolution) belonging to the A compartment as determined by Hi-C. (**c**) Fraction of 1 Mb genomic regions that either belong to the same (A → A and B → B) or switching compartment (A → B or B → A) during the transition from NES to NPC. (**d**) Same as in (**c**) but for the transition from NPC to NEU. (**e**–**g**) Distributions of normalized DSB counts in 1 Mb genomic regions belonging to the A or B compartment in NES (**a**), NPC (**b**), and NEU (**c**) cells. CPM, DSB count per million reads calculated as number of DSBs divided by number of reads times one million. *n*, number of 1 Mb genomic regions in each compartment. Asterisks: *P* value less than 0.0001 (Wilcoxon test, two-tailed). (**h**–**j**) Same as in (**e**–**g**) but comparing genomic regions that do not change (Stable) or that switch (Changing) compartment type during the transition from NES to NEU. (**k**–**m**) Same as in (**h**–**j**) but distinguishing between A/B compartments. In all the violin plots in (**e**–**m**), the violins extend from minimum to maximum and the boxplots inside each violin extend from the 25th to the 75th percentile. The horizontal bars represent the median and whiskers extend from –1.5 × IQR to + 1.5 × IQR from the closest quartile, where IQR is the inter-quartile range. The asterisks in (**e**–**m**) indicate a *P* value less than 0.01 (**), 0.001 (***) or 0.0001 (****) (Wilcoxon test, two-tailed). ns, not significant.

To further validate our datasets, we turned our attention to the 3D genome, which, in concert with transcription, has been previously implicated in the formation of endogenous DSBs^5,50^. To this end, we performed two Hi-C experiments on NES, NPC, and NEU cells (from different NES cell passages), and called A/B compartments, TADs, and chromatin loops following well-established procedures (Methods). As replicate Hi-C datasets were highly correlated (Fig. 5a), we pooled them for all subsequent analyses. We first examined the distribution of DSBs across A/B compartments^12^ (Methods). The fraction of the genome classified as belonging to the A compartment was ~50% in NES cells and progressively decreased during the differentiation towards neurons (Fig. 5b). A small fraction (~9%) of the genome switched compartment type during differentiation, with A → B transitions being more frequent (~6%) compared to B → A transitions (~3%) (Fig. 5c,d). This reflects the progressive reduction of the A compartment from NES to NEU (Fig. 5b) and is consistent with the previous finding that the interaction strength within the B compartment increases during the differentiation of mouse NPCs to neurons^51^. We then assessed the normalized DSB counts across compartments and found them to be significantly higher (*P* < 0.0001, Wilcoxon test, two-tailed) in the A compartment, in all three stages of differentiation (Fig. 5e–g). This is consistent with the notion that the A compartment corresponds to active chromatin^12^ and with our finding that DSBs are enriched in highly expressed genes in all three differentiation stages (see Fig. 3). Interestingly, the A compartment contained a higher burden of DSBs in NES compared to NEU cells, whereas the B compartment was more fragile in NEU compared to NES cells (Fig. 5e–g). To assess whether these differences might be related to A/B compartment switches during the differentiation process, we compared the normalized DSB counts between A/B compartments that either remained stable or changed during the transition from NES to NEU (Methods). The DSB burden was significantly higher (*P* < 0.0001, Wilcoxon test, two-tailed) in A/B compartments that switched during differentiation, with the largest difference observed in NES cells (Fig. 5h–j). The biggest increase in the DSB burden was observed within genomic regions that switched from B to A compartment during the transition from NES to NEU (Fig. 5k–m). Interestingly, A-compartment regions switching to the B compartment showed a slightly but significantly lower (*P* < 0.01, Wilcoxon test, two-tailed) DSB burden in NES cells compared to stable A regions, but this difference leveled out in NPC and NEU cells (Fig. 5k–m). The lowest burden of DSBs was observed in genomic regions classified as stably belonging to the B compartment, in all three stages of differentiation (Fig. 5k–m). These results suggest that the DSB burden of a given genomic region depends on its global transcriptional activity and whether the region changes compartment during differentiation.

### DSBs are enriched at TAD boundaries and around chromatin loop anchor sites

**Fig. 6:**
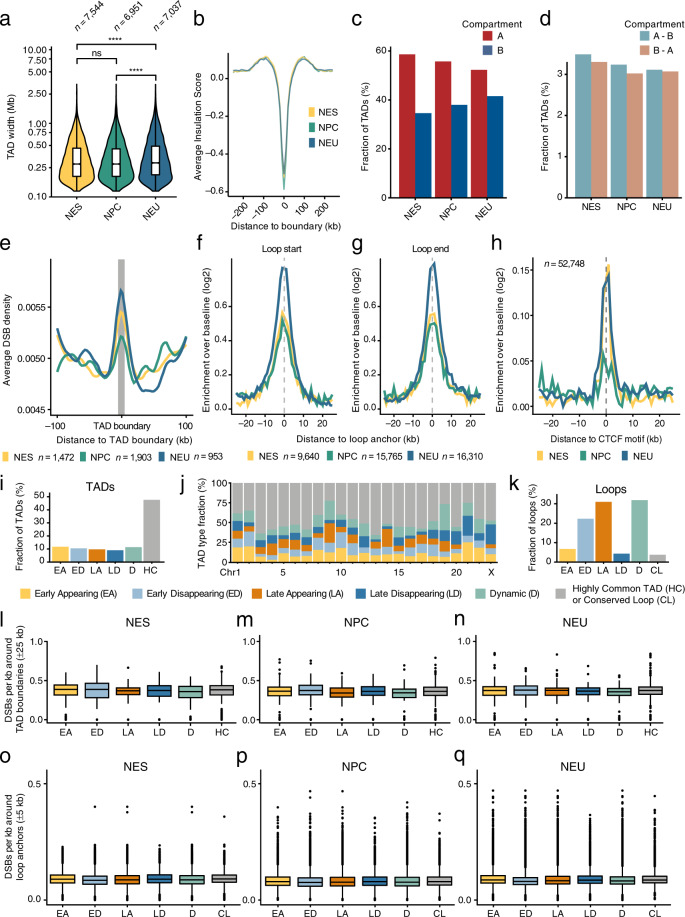
Endogenous DSBs are enriched at TAD boundaries and around chromatin loop anchors. (**a**) Distributions of the sizes of TADs identified from Hi-C datasets in NES, NPC, and NEU cells. *n*, number of TADs. *P* values are indicated above the violin plots. The violin plots extend from minimum to maximum, and the boxplots inside the violins extend from the 25th to the 75th percentile, with the horizontal bar representing the median, and whiskers extending from –1.5 × IQR to + 1.5 × IQR from the closest quartile, where IQR is the inter-quartile range. The asterisks indicate a *P* value less than 0.0001 (Wilcoxon test, two-tailed). ns, not significant. (**b**) Metaprofile of the average insulation score of TAD boundaries for each of the three differentiation stages. See Methods for how the average insulation score was calculated from the Hi-C datasets. (**c,d**) Fraction of TADs spanning genomic regions (1 Mb resolution) belonging to the same (**c**) or to a different (**d**) compartment type. (**e**) Metaprofile of the DSB density around TAD boundaries identified in NES, NPC, and NEU cells based on Hi-C data. *n*, number of TADs. (**f,g**) Metaprofiles of DSB enrichment around the upstream (**f**) and downstream (**g**) anchor site of chromatin loops identified by Hi-C in NES, NPC, and NEU cells. *n*, number of loops. (**h**) Same as in (**f,g**) but for DSB enrichment around CTCF factor binding motifs. *n*, number of CTCF motifs. (**i**) Fraction of TADs belonging to one of six categories: (1) Early Appearing (EA); (2) Early Disappearing (ED); (3) Late Appearing (LA); (4) Late Disappearing (LD); (5) Dynamic (D); and (6) Highly Common (HC), based on whether and when TADs disappear or appear during the differentiation of NES cells to NEU. See Methods for how the classification was performed. (**j**) Same as in (**i**) but separately for each chromosome. (**k**) Same as in (**i**) but for chromatin loops. Note that the last category (grey) is now referred to as Conserved Loop (CL). (**l**–**n**) Distributions of the DSB burden per kb in a genomic region of 50 kb around each TAD boundary in NES (**l**), NPC (**m**), and NEU (**n**) cells. Categories assigned as in (**i**). (**o**–**q**) Same as in (**l**–**n**) but for chromatin loops. In all the boxplots in (**l**–**q**), each boxplot extends from the 25th to the 75th percentile, the horizontal bars represent the median, and whiskers extend from –1.5 × IQR to + 1.5 × IQR from the closest quartile, where IQR is the inter-quartile range. Black dots, outliers.

Next, we examined how the endogenous DSBs detected by sBLISS in NES, NPC, and NEU cells are distributed with respect to TADs and chromatin loop anchors (Methods). The average TAD size was significantly higher (*P* < 0.01, Wilcoxon test, two-tailed) in NEU compared to NES cells, whereas the TAD insulation score remained constant (Fig. 6a,b). This is reminiscent of the larger TAD structures previously observed in mature neurons^52–54^. Consistent with the progressive expansion of the B compartment, the proportion of B-type TADs increased during the transition from NES to NEU, while the fraction of TADs overlapping different compartments slightly decreased (Fig. 6c,d). DSBs were locally enriched around TAD boundaries and chromatin loop anchors, as well around CCCTC-binding factor (CTCF) motifs in all three differentiation stages (Fig. 6e–h). These findings are in agreement with prior reports according to which chromatin loop anchors represent fragile sites where DSBs accumulate most likely as a consequence of topological stress associated with loop extrusion and active transcription of nearby genes^4,50^. We then classified TADs and chromatin loops based on their dynamics throughout the differentiation process (Fig. 6i–k and Methods). Unlike for A/B compartments, the DSB burden around TAD boundaries and chromatin loop anchors remained substantially unchanged regardless of the dynamics of these regions during differentiation (Fig. 6l–q). Collectively, these results suggest that, while endogenous DSBs form non-randomly with respect to TADs and chromatin loops, structural changes occurring in these regions during neural cell fate determination do not seem to reshape their fragility landscape.

### Endogenous DSBs form preferentially within expressed NDD risk genes

**Fig. 7:**
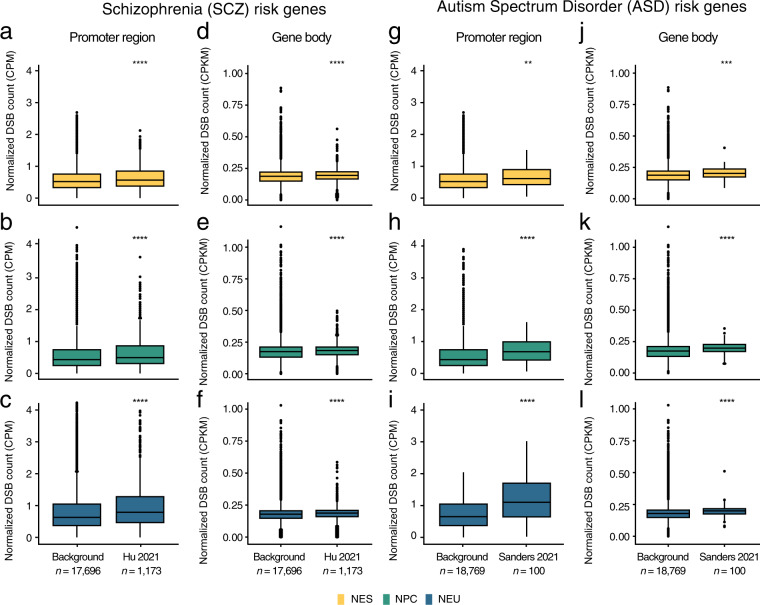
Endogenous DBSs are enriched at the promoter and along the gene body of genes associated with increased risk for schizophrenia (SCZ) and autism spectrum disorder (ASD). (**a**–**c**) Distributions of normalized DSB counts in the promoter region (from 2 kb upstream to 1 kb downstream) around the transcription start sites (TSS) of genes associated with SCZ risk (see Supplementary Data in ref. ^37^) or background genes comprising all human protein-coding genes except the examined SCZ risk genes. CPM, DSB count per million reads calculated as number of DSBs divided by number of reads times one million. *n*, number of genes in each group. (**d**–**f**) Same as in (**a**–**c**) but for normalized DSB counts along the gene body (from the first TSS of the gene to the last transcription end site). (**g**–**i**) Same as in (**a–c**) but for genes associated with ASD risk (see Table S2 in ref. ^38^). (**j**–**l**) Same as in (**h**–**j**) but for normalized DSB counts along the gene body. The asterisks in (**a**–**l**) indicate a *P* value less than 0.01 (**), 0.001 (***) or 0.0001 (****) (Wilcoxon test, two-tailed).

**Fig. 8:**
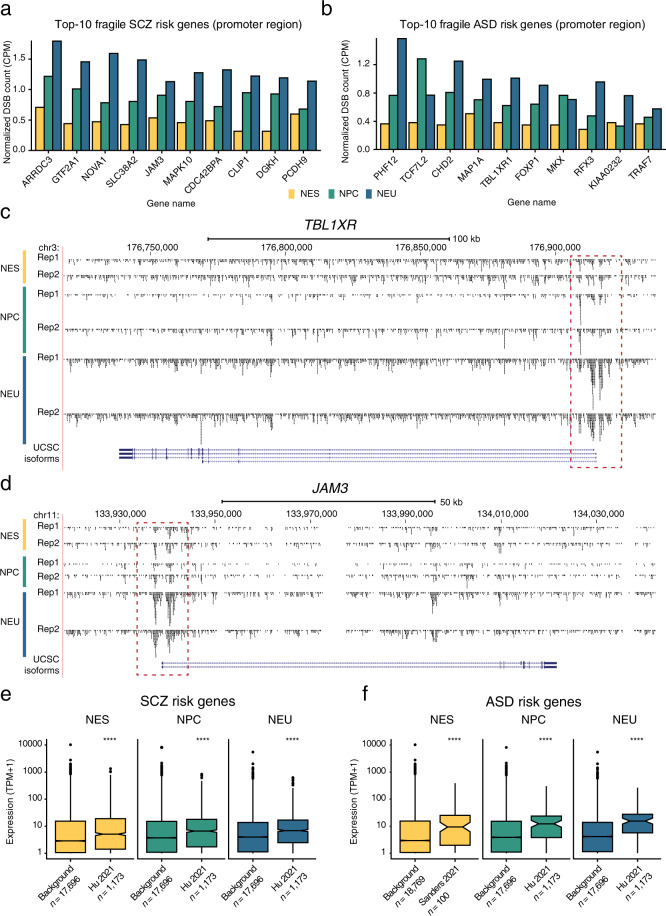
Top-fragile genes associated with increased risk for schizophrenia (SCZ) and autism spectrum disorder (ASD). (**a**) Normalized DSB counts in the promoter region (from 2 kb upstream to 1 kb downstream of the transcription start site (TSS)) for the ten most fragile genes associated with SCZ risk in NES, NPC, and NEU cells. CPKM, DSB count per kilobase per million reads calculated as number of DSBs divided by number of reads times one million divided by gene width times 1,000. (**b**) Same as in (**a**) but for the ten most fragile genes associated with ASD risk. (**c,d**) Visualization of mapped DSBs along two of the top-fragile genes shown in (**a**) and (**b**) using the *squish* option in the UCSC genome browser. The dashed red rectangles indicate the enrichment of DSBs around the TSS of the two genes. (**e**) Distributions of gene expression levels in SCZ risk genes and background genes (all human protein-coding genes except SCZ risk genes) in NES, NPC, and NEU cells. Asterisks indicate a *P* value less than 0.0001 (Wilcoxon test, two-tailed). TPM, transcripts per million. (**f**) Same as in (**e**) but for ASD risk genes.

In mouse neural stem cells, endogenous DSBs indirectly detected by HTGTS^45^ are enriched in genes whose human orthologues are associated with increased risk for schizophrenia (SCZ) and autism spectrum disorder (ASD)^6–8^. Furthermore, in human neural progenitor cells, recurrent DSB clusters identified by HTGTS map along the gene body of multiple genes involved in SCZ and ASD^9^. Therefore, to further validate our datasets, we explored whether the endogenous DSBs detected by sBLISS in human NES, NPC, and NEU cells are also enriched in genes associated with these disorders. To this end, we retrieved a list of 1,169 genes previously associated with increased SCZ risk^37^ as well as 100 genes previously associated with ASD^38^ (Supplementary Table 3). The normalized DSB counts inside the promoter region and along the gene body of these genes were significantly higher compared to the counts in the corresponding regions of all other human protein-coding genes (background), in all three cell differentiation stages analyzed (Fig. 7a–l and Methods). Furthermore, among SCZ and ASD risk genes, the DSB burden around promoters as well as along gene bodies was significantly higher in NEU compared to NES cells (Fig. 7a–l). The ten most fragile SCZ and ASD genes included genes with well-known neuronal functions as well as genes that have been previously implicated in neuro-psychiatric disorders, including *MAPK10*, *CDC42BPA*, *CLIP1* and *PCDH9* among SCZ risk genes, and *TCF7L2*, *CHD2*, *MAP1A*, *FOXP1*, *RFX3*, and *TBL1XR1* among ASD risk genes (Fig. 8a,b). In all cases, the burden of DSBs in the promoter region of these top fragile genes was consistently higher in NEU compared to NES cells (Fig. 8a–d). This suggests that, in the later stages of neural cell fate specification, these genes might experience a higher amount of transcription-related damage, possibly because of higher expression levels. Indeed, comparison with RNA-Seq data revealed that SCZ and ASD risk genes were significantly more expressed (*P* < 0.0001, Wilcoxon test, two-tailed) in NEU compared to NES cells, as well as compared to background genes (Fig. 8e,f). Altogether, these results demonstrate that, in agreement with previous observations, endogenous DSBs accumulate at gene loci associated with increased risk for NDDs, with differentiated neural cells exhibiting the highest amount of DSBs in these genes. Although most of the endogenous DSBs forming inside NDD risk genes during neurogenesis are most likely correctly repaired, it is tempting to speculate that repeated DSB repair errors occurring during neurodevelopment might change the promoter sequence of these genes and instigate pathogenic effects by affecting their expression levels and/or correct time of expression. We anticipate that the approach, tools, and datasets presented here will facilitate future studies aimed at testing this fascinating hypothesis.

## Usage Notes

We have generated what is, to our knowledge, the first atlas of endogenous DSBs that form spontaneously in an *in vitro* model mimicking the process of human neural cell fate specification. The sBLISS BED files that we provide (see Data Records) can be readily used to visualize the pattern of DSBs along any gene of interest using freely available tools such as the UCSC Genome Browser (https://genome.ucsc.edu/) or the Integrative Genomics Viewer (https://software.broadinstitute.org/software/igv/). Moreover, the accompanying RNA-Seq and Hi-C datasets represent a valuable resource that can be further mined to investigate the interplay between 3D genome organization and transcriptional dynamics during neural cell fate specification. We provide all the custom scripts and relative documentation needed to reproduce the pre-processing of sBLISS, RNA-Seq, and Hi-C datasets as well as all the analyses described here. One limitation of our study is that it was conducted on an *in vitro* model system that most likely only partially recapitulates the landscape of genome fragility in the nervous system. Hence, future studies applying sBLISS to more complex *in vitro* systems, such as brain organoids, as well as to nuclei extracted directly from brain tissue are needed to fully characterize the landscape of genome fragility in the developing and adult brain.

## Supplementary information

Supplementary Table 1

Supplementary Table 2

Supplementary Table 3

## Data Availability

Custom scripts used for processing the sBLISS, RNA-Seq, and Hi-C datasets are available at https://github.com/BiCroLab/NatSciData_Neuro. Custom scripts used to analyze the immunofluorescence images are available at https://github.com/elgw/sci_data_20220516.
